# Preparation, amino acid composition, and in Vitro antioxidant activity of okra seed meal protein hydrolysates

**DOI:** 10.1002/fsn3.2263

**Published:** 2021-04-02

**Authors:** Hongliang Yao, Jiani Yang, Jiajia Zhan, Qu Lu, Min Su, Yaojiong Jiang

**Affiliations:** ^1^ Department of Food Science Jinling Institute of Technology Nanjing Jiangsu Province China

**Keywords:** amino acid composition, antioxidant capacity, Okra seed, orthogonal experiment, protein hydrolysates, response surface methodology (RSM)

## Abstract

To improve the utilization of okra seed, acidic and enzymatic hydrolyses of producing protein hydrolysates were respectively optimized by orthogonal experiment and response surface methodology using the degree of hydrolysis (DH) as evaluating index. Amino acid composition and antioxidant capacity in vitro of two kinds of hydrolysates were both analyzed. The degree of acidic hydrolysis was 58.53 ± 1.92% under the following optimized condition: hydrolyzing time 40 hr, temperature 95°C, ratio of acid solution to okra seed meal (OSM) powder was 5:1 (V:W/ml:g), and hydrochloric acid concentration was 18% (W/W). The degree of enzymatic hydrolysis was 16.26 ± 0.56% under the optimized condition: hydrolyzing time 8.20 hr, ratio of buffer to OSM powder was 10:1, and enzyme dosage was 3,100 International Units (IU) g^−1^. Enzymatic hydrolysates had a fuller range of amino acids and antioxidant capacity than acidic hydrolysates. The results provide technical support for the expansion of okra seed utilization.

## Graphical abstract

Acidic and enzymatic protein hydrolysates were produced from okra seed by optimized hydrolysis processes. Enzymatic protein hydrolysates provided fuller range amino acid and more antioxidant capacity than acidic protein hydrolysates from okra seed meal.

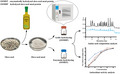

## 1 INTRODUCTION

Okra (*Abelmoschus esculentus* (L.) Moench) belongs to *Malvaceae* family which is a kind of plant originated in Africa (Ames and Macleod, 1990). Nowadays, it is widely cultured in tropics, subtropics, and temperate regions for its immature seed pods which are consumed as a vegetable (Rizwan et al., 2020; Bawa and Badrie, 2016; Camciuc et al., 1998). In the past decade, different strains of okra have been widely cultured in the South and North of China (Nie et al., 2019). Okra is a healthy food because it is rich in dietary fiber, potassium, magnesium, vitamin C, folate, and bioactive components, such as flavonoids (Bawa and Badrie, 2016). It is also used in folk medicine for preventing liver damage, and abating gastric inflammation (Peng et al., 2016). Dozens of articles indicated that okra possesses bioactive functions, such as antioxidant activity, anti‐inflammatory and immune‐regulatory effects, antibacterial activity, gastro protective effect, and so on (Islam, 2019).

Studies published dozens of years ago have already confirmed that okra seed is a good source of protein and oil (Martin and Ruberte, 1979; Karakoltsidis & Constantinides, 1975). Recently, okra seed lipid extraction and its fatty acid composition were researched (Geórgia et al., 2012; Jarret et al., 2011; Mariod et al., 2011; Anwar et al., 2010), and okra seed oil was also considered as a potential bioenergy (Moosavi et al., 2018; Sandlin, 2011). Moreover, okra seed was added in regional traditional food (Akingbala et al., 2003; Aminigo and Akingbala, 2004; Ray et al., 2017) or family food (Xu et al., 2020) to increase crude protein content, high viscosities, and bio‐function. Despite these facts, okra seed is yet to be fully exploited in food industry as a source of vegetable protein (Liu et al., 2019; Oyelade et al., 2003).

As an important component in dried matured okra seed, the crude protein is about 20% normally (Geórgia et al., 2012), even is up to 45% after defatting (Adelakun et al., 2009), indicating that it could be a good source of hydrolyzed vegetable protein (HVP) (Jeon et al., 2020). Hydrolyzed protein has been traditionally used as a food ingredient in East Asian countries, such as China, Japan, and Indonesia, and was later researched and applied in food industry. HVP is commonly produced using vegetable protein sources, such as soybean, wheat, and maize as material (Aaslyng et al., 1998). Acidic hydrolysis was earlier researched as the traditional method (Velíšek et al., 1993), afterward the enzymatic hydrolysis method was studied (Aaslyng, Poll et al., 1999; Sinha et al., 2006). The products are respectively referred to as HVP and enzymatically hydrolyzed vegetable protein (EVP).

Although a study on preparation of antioxidant peptide from okra seed was reported, there is currently not much information available in the field of protein hydrolysates using okra seed as starting material (Guo et al., 2018). In this study, the optimization of acidic hydrolysis and enzymatic hydrolysis, analysis of amino acid composition and antioxidant capacity in vitro of two kinds of protein hydrolysates will be researched, with the aim of supporting theoretical basis for extending further processing for okra seed such as HVP or EVP.

## 2 MATERIALS AND METHODS

### 2.1 Okra seed meal and reagents

Okra seeds were purchased from a local farm then defatted refer to our previous research (Yao et al., 2019). After drying in oven at 60°C, the OSM powder was ground and then stored in fridge by vacuum packing. Its crude protein content was 36.9 ± 0.2% detected by Kjeldahl method.

2, 2‐Azino‐bis [3‐ethylbenzthiazoline‐6‐sulfonic acid]‐diammonium salt (ABTS) was purchased from Shanghai Aladdin Bio‐Chem Technology Co., Ltd. Neutrase, Papain, and Alkaline protease were purchased from Shanghai Yuanye Bio‐Technology Co., Ltd. Other reagents were purchased from Nanjing Shoude Chemicals Ltd. All the above reagents were of analytical grade.

### 2.2 Acidic hydrolysis optimization on okra seed

Hydrolyzed okra seed meal protein (OSMHP) was obtained by hydrochloric acid hydrolysis using previously published methods (Aaslyng, Poll et al., 1999; Aaslying, Larsen et al., 1999) with modification. One gram OSM powder was mixed with hydrochloric acid whose concentration ranged from 12% to 20% in closed glass bottle; then, the mixture was blended and heated in oven with a specified temperature ranged from 90 to 105°C for a specified time ranged from 20 to 48 hr. After cooling to room temperature, the mixture was neutralized to pH 7.0 with 4 M NaOH; then, the supernatant was collected after centrifuging (23,120 ***g***, 10 min, 4°C); afterward, it was adjusted to constant volume in a volumetric flask using pure water for DH detecting. Free amino acid nitrogen amount was detected by potentiometric titration.

(1) $$\text{DH},\%=\frac{\text{free amino acid nitrogen amount in hydrolysate}}{\text{total nitrogen amount in okra seed meal sample}}\times 100\%$$

Single‐factor experiment was carried out for studying the effect of hydrolyzing condition on degree of hydrolysis. The single factors included hydrolyzing time (A: 20, 24, 28, 32, 36, 40, 44, 48 hr), temperature (B: 90–105°C), ratio of acid solution to OSM powder (C: 4–8:1, V/W: mL/g), and hydrochloric acid concentration (D: 12%–20%, W/W). Based on the single‐factor experiment, further orthogonal experiment design with four factors three levels was employed for acidic hydrolysis optimization.

### 2.3 Enzymatic hydrolysis optimization on okra seed

The enzymatical hydrolyzing was referred to the method published previously (Aaslyng, Poll et al., 1999, Aaslying, Larsen et al., 1999) and modified to produce enzymatically hydrolyzed okra seed meal protein (OSMEP). One gram okra seed meal powder was mixed with buffer solution containing protease in closed glass bottle; then, the mixture was blended and incubated for a specified time ranged from 5 to 10 hr. After hydrolyzing, the enzyme was deactivated by heat treatment in boiling water for 10 min; after cooling to room temperature, the supernatant was collected after centrifuging (23,120 ***g***, 10 min, 4°C); afterward, it was adjusted to constant volume in a volumetric flask using pure water for further detecting. Then, DH was calculated using Equation (1).

Under condition of the respective optimized pH value and temperature referring to the commercial enzyme manual, three kinds of protease including Neutrase, Papain, and Alkaline protease were evaluated for further research with respect to the degree of hydrolysis on okra seed. Research articles already reported the most important factors effect enzymatic hydrolysis are pH value, hydrolyzing temperature, enzyme concentration, enzyme dosage, and hydrolyzing time; these factors often interact with one another (Chabeaud et al., 2009; Meyabadi & Dadashian, 2012). The optimized pH value and optimized temperature of the enzyme we used were specified. Hence, hydrolyzing time (A: 5–10 hr), ratio of buffer to OSM powder (B: 10–50:1, V: W/mL:g), and enzyme dosage (C: 1–6 × 10^3^ IU/g) were adopted as factors in single‐factor experiment. Then based on the single‐factor experiment, three‐factor experimental design was employed for optimizing enzymatic hydrolyzing using Box–Behnken design of response surface method (RSM). Three independent variables were coded at three levels (−1, 0, 1) on DH, which resulted in a 17‐run experiment.

### 2.4 Amino acid composition analysis on OSMHP and OSMEP

Reactions that were 15 times bigger than before were employed for producing hydrolyzed okra seed meal protein (OSMHP) and enzymatically hydrolyzed okra seed meal protein (OSMEP), respectively, under the optimized condition we researched in this paper. Each supernatant was adjusted to 250 ml finally for amino acid composition analysis and antioxidant activity analysis in vitro. The final hydrolysate solutions were used for amino acid composition analysis directly without dilution, while they were diluted to different concentration between 0.01 ~ 0.5 mg/ml with a gradient of 0.05 mg/ml for antioxidant activity analysis in vitro.

A 16 amino acid analysis was carried out by automatic amino acid analyzer (Sykam S‐433D, Germany) refers to national standard (National Health and Family Planning Commission of PRC et al. 2017). Tryptophan was detected by HPLC‐FLD method (Agilent 1,260, USA) refers to national standard (State Administration for Market Regulation, 2018).

### 2.5 Antioxidant activity analysis in vitro

Compared to ascorbic acid, antioxidant activity in vitro of OSMHP and OSMEP were measured using hydroxyl radical (OH·) scavenging capacity and ABTS free radical (ABTS·) scavenging capacity as indicator. Also, the 50% effective concentration on free radical scavenging (EC_50_) was analyzed by IBM SPSS 22.0 software.

#### 2.5.1 Hydroxyl free radical scavenging assay

Refer to the method (Nie et al., 2017) with modification, hydroxyl free radical scavenging assay was as follows: Four centrifuge tubes were marked with *A*_blank_, *A*_0_, *A*_x_, and *A*_x0_; then, different reagents were added in order which was shown in Table 1, then incubated at 37°C for 15 min; after cooling to room temperature, all mixtures were detected by spectrometer at 510 nm after zero setting with blank solution which marked *A*_blank_; then, the scavenging ratios were calculated according to Equation (2).

(2) $$\text{OH}\cdot \text{scavenging ratio}=\left(1‐\frac{A_{X}‐A_{X_{0}}}{A_{0}}\right)\times 100\%$$

**TABLE 1:** Reagents adding order and volume ml

Reagent	*A*_x_	*A*_x0_	*A*_0_	*A*_blank_
9 mmol/L FeSO_4_	1	1	1	1
9 mmol/L Salicylic acid ethanol solution	1	1	1	1
Sample (OSMHP, OSMEP, or ascorbic acid)	6	6	/	/
Purified water	/	1	6	7
Hydrogen peroxide	1	/	1	/

Abbreviations: OSMEP, enzymatically hydrolyzed okra seed meal protein; OSMHP, hydrolyzed okra seed meal protein.

#### 2.5.2 ABTS free radical scavenging assay

**FIGURE 1:**
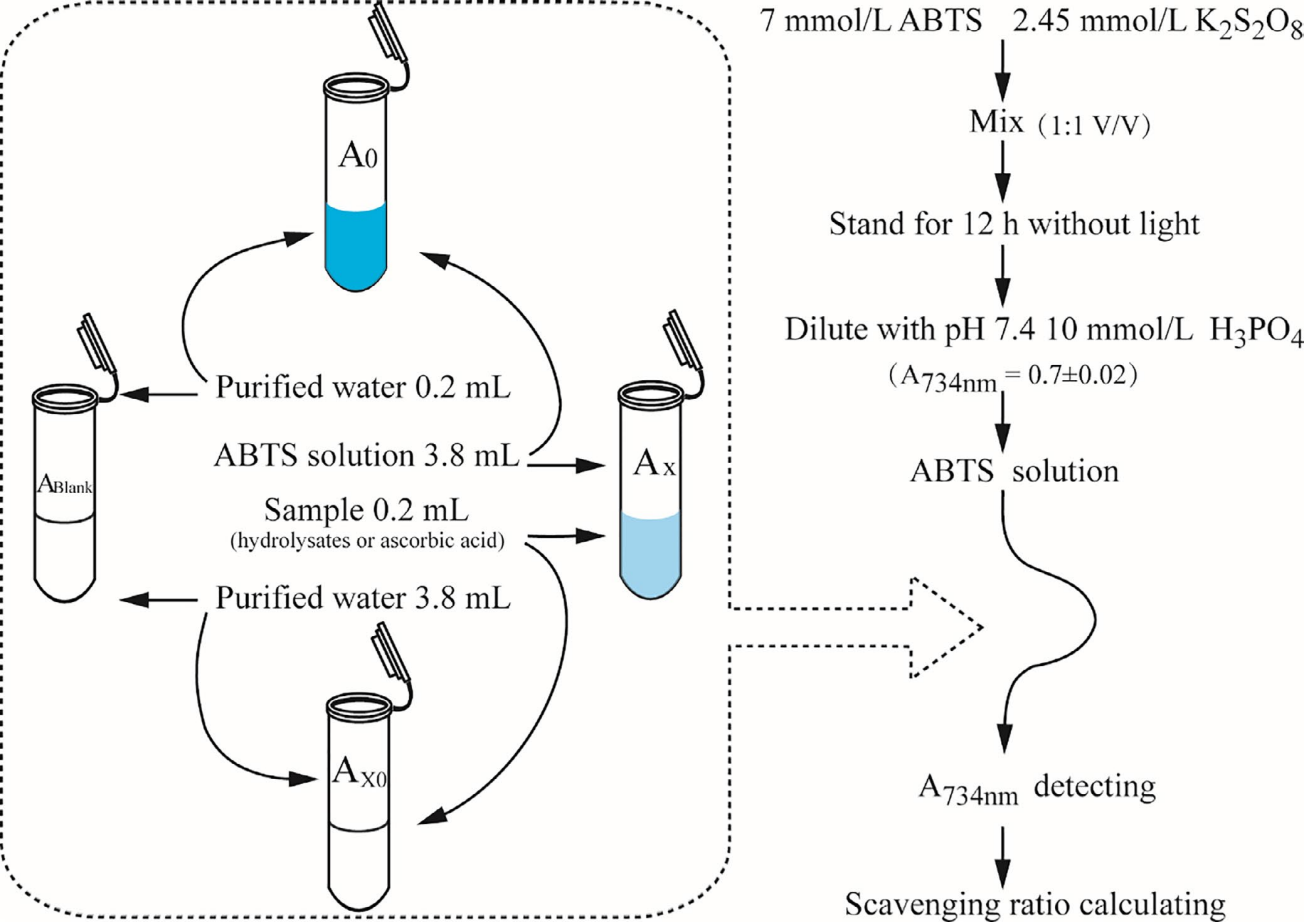
The detecting procedure of ABTS (2, 2‐Azino‐bis (3‐ethylbenzthiazoline‐6‐sulfonic acid)) free radical scavenging capacity analysis

ABTS free radical assay was referred to the published method (Xu et al., 2018) with modification which was absolute fit in this study. The method was shown in Figure 1, and the scavenging ratios were calculated according to Equation (3).

(3) $$\text{ABTS}\cdot \;\text{scavenging ratio}=\left(1‐\frac{A_{X}‐A_{X_{0}}}{A_{0}}\right)\times 100\%$$

### 2.6 Statistical analysis

The IBM SPSS 22.0 software was employed in this research. One‐way ANOVA post hoc multiple comparisons were employed in single‐factor experiment, and general linear model analysis was used in orthogonal experiment for acidic hydrolyzing optimization. All experiments were conducted in triplicates. The result was shown as mean ± standard deviation.

## 3 RESULT AND DISCUSSION

### 3.1 Acidic hydrolysis optimization on okra seed

**FIGURE 2:**
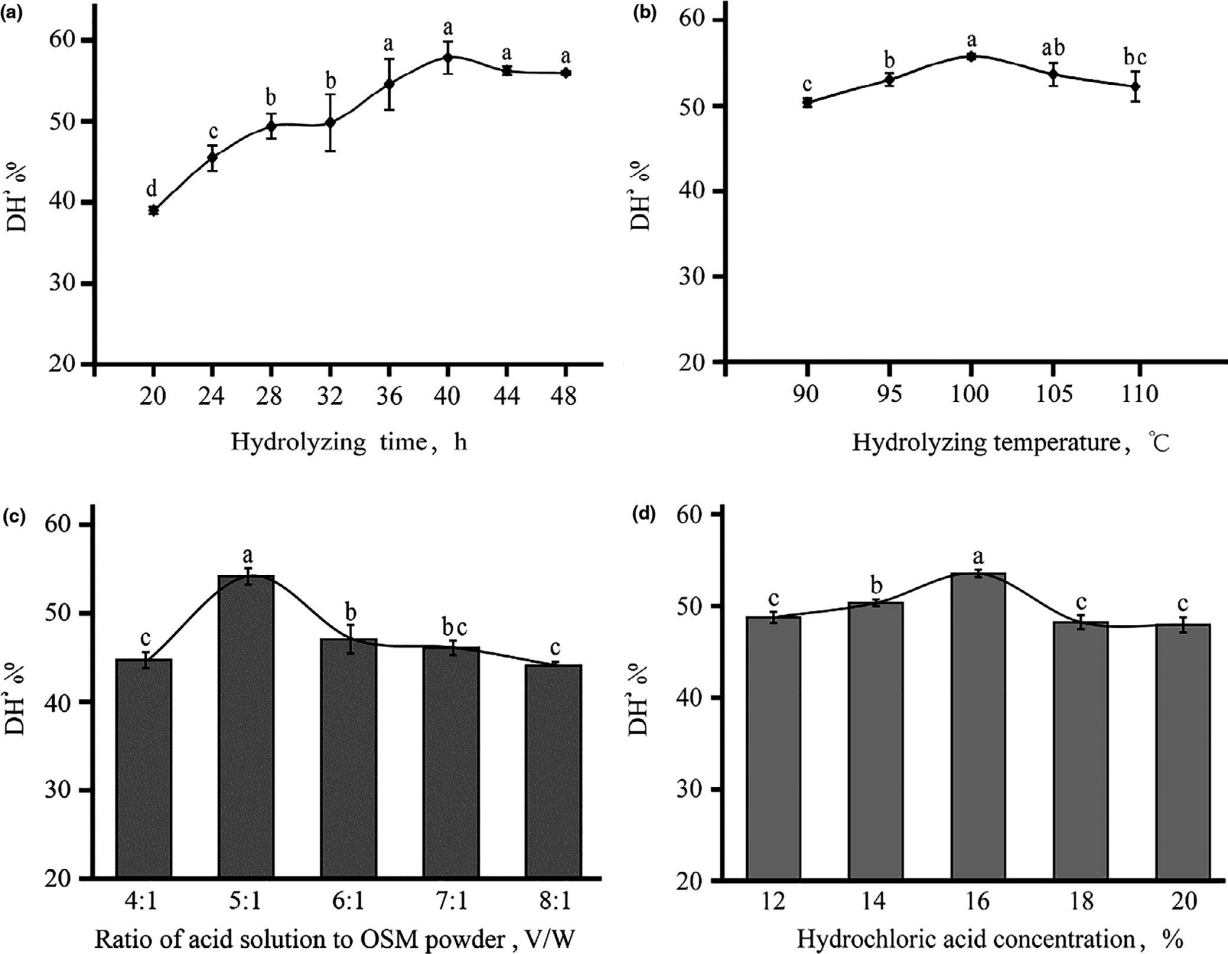
Single‐factor experiment for acid hydrolyzing. (a) Effects of hydrolyzing time on degree of hydrolysis (DH); (b) effects of hydrolyzing temperature on degree of hydrolysis (DH); (c) effects of ratio of acid solution to OSM powder on degree of hydrolysis (DH); and (d) effects of hydrochloric acid concentration on degree of hydrolysis (DH). Different small letter means significant different in Figure 2, *p* =.05

As can be seen from Figure 2a–d, the DH increased with the extension of hydrolyzing time, then reached the highest after 40 hr of hydrolyzing. DH was maintained at a stable level even though the hydrolysis was extended. Actually, it can be seen that the hydrolyzing time did not affect DH significantly after 36 hr.

DH increased with the raising of hydrolyzing temperature, then reached an inflection point at 100°C. Moreover, the reaction mixture started boiling once the hydrolyzing temperature reached to 110°C, thus limiting the ability to extent the hydrolysis temperature. DH also increased with an increase in the ratio of acid solution to OSM powder and hydrochloric acid concentration, then reached an inflection. A previous study noted that hydrolyzing protein in hot acid, normally for 24 hr, leads to peptide bond cleavage then amino acid degradation (Darragh et al.,^1996^), it means that adequate or optimized hydrolysis time, hydrochloric acid solution volume and concentration can ensure the hydrolysis efficiency while too much will result in a decrease. This is similar to the results obtained in this study.

According to the single experiment result and analysis, the factors and levels were selected for further orthogonal experiment as shown in Table 2. The orthogonal experiment results are shown in Table 3. General linear model univariate statistical analysis (Table 4) showed that hydrolyzing time, hydrolyzing temperature, and ratio of acid solution to OSM powder all significantly affected the DH (*p* < .01), while hydrochloric acid concentration did not (*p* > .05).

**TABLE 2:** Factors and levels in orthogonal experiment design

Level	A: hydrolyzing time (h)	B: hydrolyzing temperature (°C)	C: ratio of acid solution to OSM powder (V :W/ml: g)	D: hydrochloric acid concentration (%, W/W)
1	36	95	4:1	14
2	40	100	5:1	16
3	44	105	6:1	18

**TABLE 3:** Orthogonal experiment result of okra seed meal hydrolysis

Run	A: hydrolyzing time (h)	B: hydrolyzing temperature (°C)	C: ratio of acid solution to OSM powder (V: W/ml: g)	D: hydrochloric acid concentration (%, W/W)	Degree of hydrolysis (DH) (%)
1	36 (1)	95 (1)	4:1 (1)	14% (1)	54.08
2	36 (1)	100 (2)	5:1 (2)	16% (2)	53.39
3	36 (1)	105 (3)	6:1 (3)	18% (3)	47.27
**4**	40 (2)	95 (1)	5:1 (2)	18% (3)	58.53
5	40 (2)	100 (2)	6:1 (3)	14% (1)	51.91
6	40 (2)	105 (3)	4:1 (1)	16% (2)	45.10
7	44 (3)	95 (1)	6:1 (3)	16% (2)	47.77
8	44 (3)	100 (2)	4:1 (1)	18% (3)	47.37
9	44 (3)	105 (3)	5:1 (2)	14% (1)	45.00
K_1_	154.7	160.4	146.6	151.0	
K_2_	155.6	152.7	156.9	146.3	
K_3_	140.1	137.4	147.0	153.2	
R	15.5	23.0	10.3	6.9	

**TABLE 4:** General linear model analysis for orthogonal experiment

Source	Sum of squares	*df*	Mean square	*F* value	Significance
Model	68,146.903	9	7,571.878	1,400.087	0.000
Hydrolyzing time	149.603	2	74.801	13.831	0.000
Hydrolyzing temperature	273.276	2	136.638	25.265	0.000
Raito of acid solution to OSM powder	69.201	2	34.600	6.398	0.008
Hydrochloric acid concentration	24.701	2	12.380	2.289	0.130
Error	97.347	18	5.408		
Total	0.12	27			

According to the orthogonal experiment result, the optimized permutation was A_2_B_1_C_2_D_3_ which is similar to what is presented in Table 3. Therefore, the optimized acidic hydrolyzing parameter is shown as follows: hydrolyzing time 40 hr, hydrolyzing temperature 95°C, ratio of acid solution to OSM powder 5:1 (V: W/ml: g), and hydrochloric acid concentration was 18% (W/W). Under this condition, DH reached its highest at 58.53 ± 1.92%. Tan et al. (2019) used soybean meal to produce acidic hydrolysates, and the DH was 30%–50% under the condition: hydrolysis temperature 110°C, hydrolysis time 5 hr, hydrochloric acid concentration 3 mol/L, and the ratio of acid solution to soybean meal was 5:1. In another study on acidic hydrolysis using soybean meal as material, the DH was as high as 93.5% under the optimized condition: hydrolysis temperature 120°C, hydrolysis time 6 hr, sulfuric acid concentration 20% (W/W), and the ratio of acid solution to soybean meal was 5:1 (Bai et al., 2005).

Therefore, in order to obtain high degree of hydrolysis, relatively harsh conditions are needed, such as high hydrolysis temperature, strong and high acid concentration, and long hydrolysis time, while the ratio of acid solution to material can be fixed at 5:1.

### 3.2 Enzymatic hydrolysis optimization on okra seed

**FIGURE 3:**
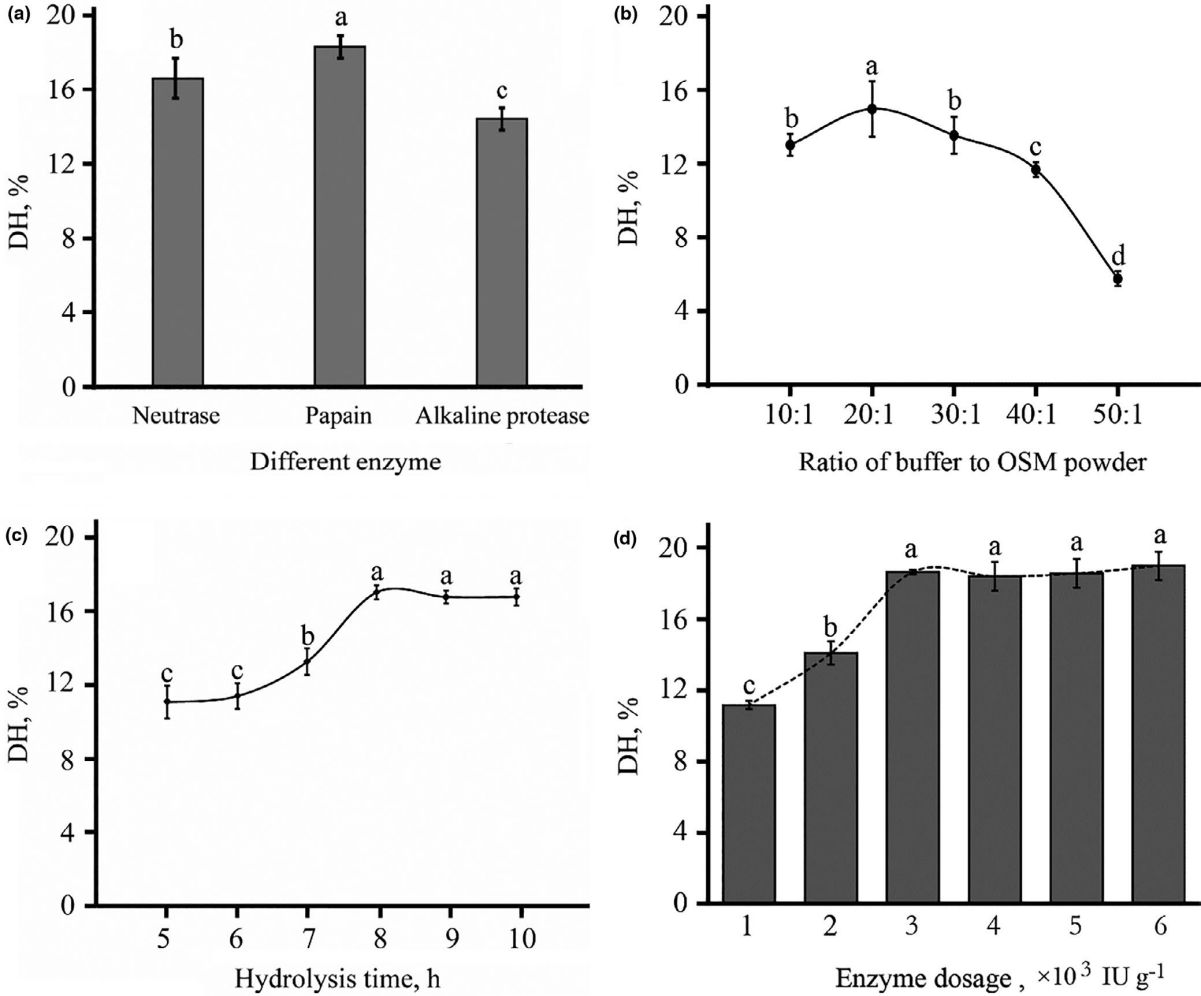
The result of single‐factor experiment for enzymatically hydrolyzing. (a) Selection of enzyme; (b) effects of ratio of buffer to OSM powder on degree of hydrolysis (DH); (c) effects of hydrolyzing time on degree of hydrolysis (DH); and (d) effects of enzyme dosage on degree of hydrolysis (DH). Different small letter means significant different in Figure 3, *p* = .0

Papain was selected for further enzymatical hydrolyzing research (Figure 3a). DH increased to an inflection point then decreased sharply when the ratio of buffer to OSM powder was raised (Figure 3b). In the case of constant substrate, enough buffer can promote the enzymatic reaction via increasing interaction between enzyme and substrate while too much buffer can decrease the enzyme concentration thereby weakening the enzymatic efficiency.

There was an initial increase in DH until it reached a stable level with the extension of hydrolysis time (Figure 3c). This was similar to the effects of enzyme dosage on DH (Figure 3d). The results indicated that okra seed meal protein was adequately hydrolyzed by papain. Adequate hydrolyzing time and enzyme dosage can ensure complete enzymatic reaction, once the reaction reached saturation, neither more hydrolyzing time nor enzyme dosage had positive effects.

During the process of amylase hydrolysis of corn starch, the degree of hydrolysis increased sharply within 16 hr; a slow enhancement was noticed from 16 hr to 24 hr, and reached the maximum after 24 hr. The degree of hydrolysis reached to a saturated level with the increase of enzyme dosage (Zhang & Jin, 2011). In addition to this research, Xu and his colleagues reported that the degree of hydrolysis on casein by Neutrase cannot be influenced when the hydrolysis time is more than 4 hr (Xu et al., 2014). These were consistent with our result.

According to the single experiment result and analysis, the factors and levels were selected for further experiment designed by RSM as shown in Table 5. The ANOVA for response surface quadratic model was presented in Table 6.

**TABLE 5:** Coded values of factors and result of optimization experiment using Box–Behnken design of response surface methodology (RSM)

Run	A: hydrolyzing time (h)	B: ratio of buffer to OSM powder (V: W/ml:g)	C: enzyme additive amount (×103 U/g substrate)	Degree of hydrolysis (DH) (%)
1	0 (8)	0 (20)	0 (3)	15.52
2	−1 (7)	0	1 (4)	10.15
3	0	1 (30)	−1 (2)	12.27
4	1 (9)	1	0	12.98
5	0	−1 (10)	1	15.36
6	0	0	0	15.65
7	0	0	0	15.60
8	0	0	0	14.98
9	1	−1	0	16.50
10	−1	−1	0	12.15
11	1	0	−1	11.76
12	0	1	1	14.13
13	−1	0	−1	9.85
14	−1	1	0	9.43
15	1	−1	1	10.29
16	0	−1	−1	12.80
17	0	0	0	17.56

**TABLE 6:** ANOVA for response surface quadratic model

Source	Sum of squares	*df*	Mean square	*F* value	Prob > F	
Model	88.22	9	9.80	4.70	0.0267	Significant
A	12.38	1	12.38	5.94	0.0449	
B	8.00	1	8.00	3.84	0.0909	
C	1.32	1	1.32	0.63	0.4521	
AB	0.16	1	0.16	0.077	0.7897	
AC	0.78	1	0.78	0.38	0.5592	
BC	0.12	1	0.12	0.059	0.8154	
A^2^	40.78	1	40.78	19.57	0.0031	
B^2^	0.0009792	1	0.0009792	0.00047	0.9833	
C^2^	21.07	1	21.07	10.12	0.0155	
Residual	14.58	7	2.08			
Lack of fit	10.69	3	3.56	3.66	0.1210	Not significant
Pure error	3.89	4	0.97			
Cor total	102.81	16				

Note

*R* ^2^ = 0.858, *R* ^2^_Adj_ = 0.6757, *df*: degree of freedom, Prob: probability.

By using multiple regression analysis, a final equation was obtained as follows: DH, %=15.86 + 1.24A − 1.00B + 0.41C − 0.20AB − 0.44AC − 0.17BC − 3.11A^2^ + 0.015B^2^ − 2.24C^2^. A, B, and C were the coded variables for hydrolyzing time, ratio of buffer to OSM powder, and enzyme dosage, respectively. The p value of the model was 0.0267, indicating that the fitness of the model was significant. The lack of fit value was 0.1210, indicating that it was not significant relative to the pure error. The *R* ^2^ and *R* ^2^_Adj_ are 0.8581 and 0.6757, respectively. The data in Table 6 indicated that the linear coefficient A and quadratic term coefficient A^2^, C^2^ significantly affected the DH, while the interaction effects of each two independent variables were all not significant.

**FIGURE 4:**
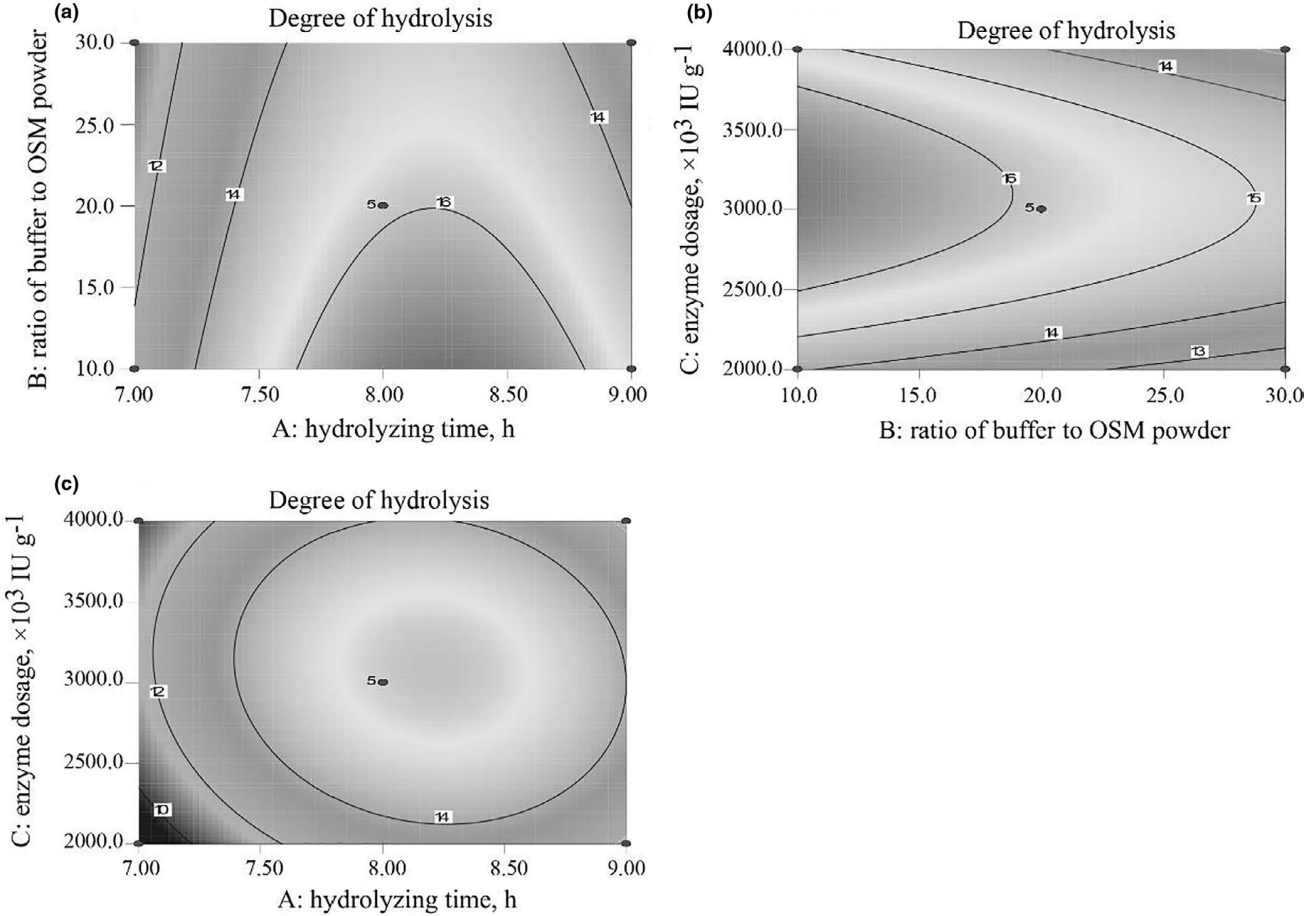
Contour plots of the combined effects of (a) hydrolyzing time and ratio of buffer to OSM powder; (b) ratio of buffer to OSM powder and enzyme dosage; and (c) hydrolyzing time and enzyme dosage, on degree of hydrolysis (DH)

In order to understand the main effect and interaction between the independent variables well, contour plots were presented in Figure 4. When two variables were investigated, the other one was fixed in its middle value level. Contour plots in Figure 4(a) showed that DH increased first then decreased by increasing hydrolyzing time while it decreased slightly with the increasing ratio of buffer to OSM powder. Contour plots in Figure 4(b) showed that DH increased first then decreased by increasing enzyme additive amount while it decreased with the increasing ratio of buffer to OSM powder. Contour plots in Figure 4(c) showed that DH increased first then decreased by increasing hydrolysis time and also increased first then decreased by increasing enzyme additive amount. An optimized solution for hydrolysis was selected as follows: hydrolyzing time 8.22 hr, ratio of buffer to OSM powder was 10:1 and enzyme additive amount was 3,106.67 U/g, and the predicated DH was 17.07%. Then, an experiment was carried out to verify the model under the following condition: hydrolyze time 8.20 hr, ratio of buffer to OSM powder was 10:1 and enzyme additive amount was 3,100 IU/g, the pH value and hydrolyzing temperature were 7.0 and 55°C, respectively, which were already specified. The actual DH reached was 16.26 ± 0.56% (*n* = 3) which was roughly consistent with the predicted value.

Compared with the harsh conditions of acid hydrolysis, the enzymatic hydrolysis conditions are much milder, so the DH of enzymatic hydrolysis cannot reach the level of the former. Xu and his colleagues (Xu et al., 2019) used soybean meal to produce hydrolyzed peptides, and the DH was 16.36% under the optimized condition by combined enzymatic hydrolysis including alkaline protease, Neutrase, and debittering enzymolysis. In another study, rapeseed meal was enzymatically hydrolyzed using microwave assistance for a period of 7 min and the DH obtained was 12.57% under the optimized condition (Li et al., 2010).

### 3.3 Amino acid composition analysis on OSMHP and OSMEP

**FIGURE 5:**
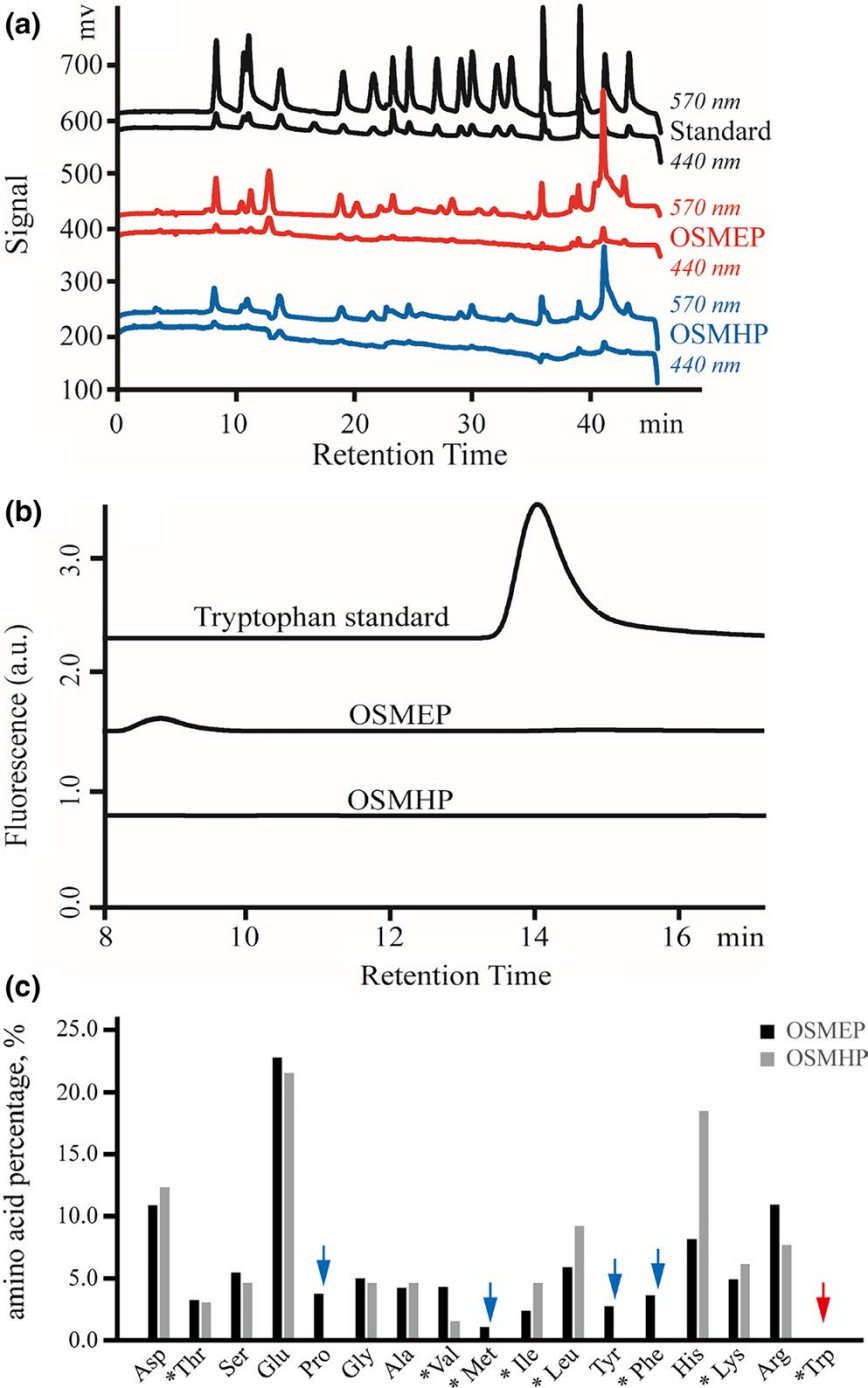
The result of amino acid analysis for enzymatically hydrolyzed okra seed meal protein (OSMEP) and hydrolyzed okra seed meal protein (OSMHP). (a) Amino acid analyzer chromatograph; (b) HPLC chromatograph for tryptophan; and (c) comparison of amino acid (*means essential amino acid)

The amino acid composition of OSMHP and OSMEP detection results is shown in Figure 5a–c. Essential amino acid percentage of OSMEP and OSMHP was 25.5% and 24.6%, respectively (Table 7); although this important value for protein hydrolysates nutrition was similar, the range of amino acid in OSMEP was fuller than that of OSMHP which lacked of Pro, Met, Tyr, and Phe. This deficiency most probably due to the damage on amino acid from strong acid during the hydrolyzing (Darragh et al.,^1996^).

**TABLE 7:** Amino acid percentage composition of OSMEP and OSMHP

Amino acid	Percentage (%)	
	OSMEP	OSMHP
Asp	10.9	12.3
Thr^a^	3.3	3.1
Ser	5.5	4.6
Glu	22.8	21.5
Pro^b^	3.7	ND
Gly	5.0	4.6
Ala^b^	4.2	4.6
Val^a^, ^b^	4.3	1.5
Met^a^, ^b^	1.1	ND
Ile^a^, ^b^	2.4	4.6
Leu^a^, ^b^	5.9	9.2
Tyr	2.7	ND
Phe^a^, ^b^	3.6	ND
His	8.1	18.5
Lys^a^	4.9	6.2
Arg	10.9	7.7
Trp^a^, ^b^	ND	ND
Essential amino acid	25.5	24.6
Hydrophobic amino acid	25.2	20.0

Note

ND, none detected.

^a^ Essential amino acid.

^b^ Hydrophobic amino acid.

In previous related research, it was found that essential amino acid and arginine percentage of soybean meal acid hydrolysate was 30.2% and about 4.5% (Aaslyng et al., 1998), while that of soybean meal enzymatic hydrolysate was 58.5% and undetected, respectively (Wu & Cadwallader, 2002).

Obviously, the percentage of essential amino acids in OSM Hydrolysates was much lower than those in soybean meal hydrolysates, while the content of arginine was much higher than that in soybean meal hydrolysates. Although arginine is not an essential amino acid, it plays an important role in physiological function, metabolism, and nutrition (Liang & Yang, 2020). The result that the OSMEP is rich in arginine provides a theoretical basis for its development and utilization.

Previous researches have indicated that tryptophan was present in both okra seed and pod (Balasubramanian & Sadasivam, 1987; Roy et al., 2014), and tryptophan was destroyed during acid hydrolysis (Karakoltsidis & Constantinides, 1975; Tsugita & Scheffler, 1982), thereby its deficiency in OSMHP is understandable. While the absence of tryptophan in OSMEP means it was destroyed during enzymatic hydrolysis. To confirm this conjecture, a further research beyond this article is necessary because little published information in this field was found.

### 3.4 Antioxidant capacity analysis in vitro

**FIGURE 6:**
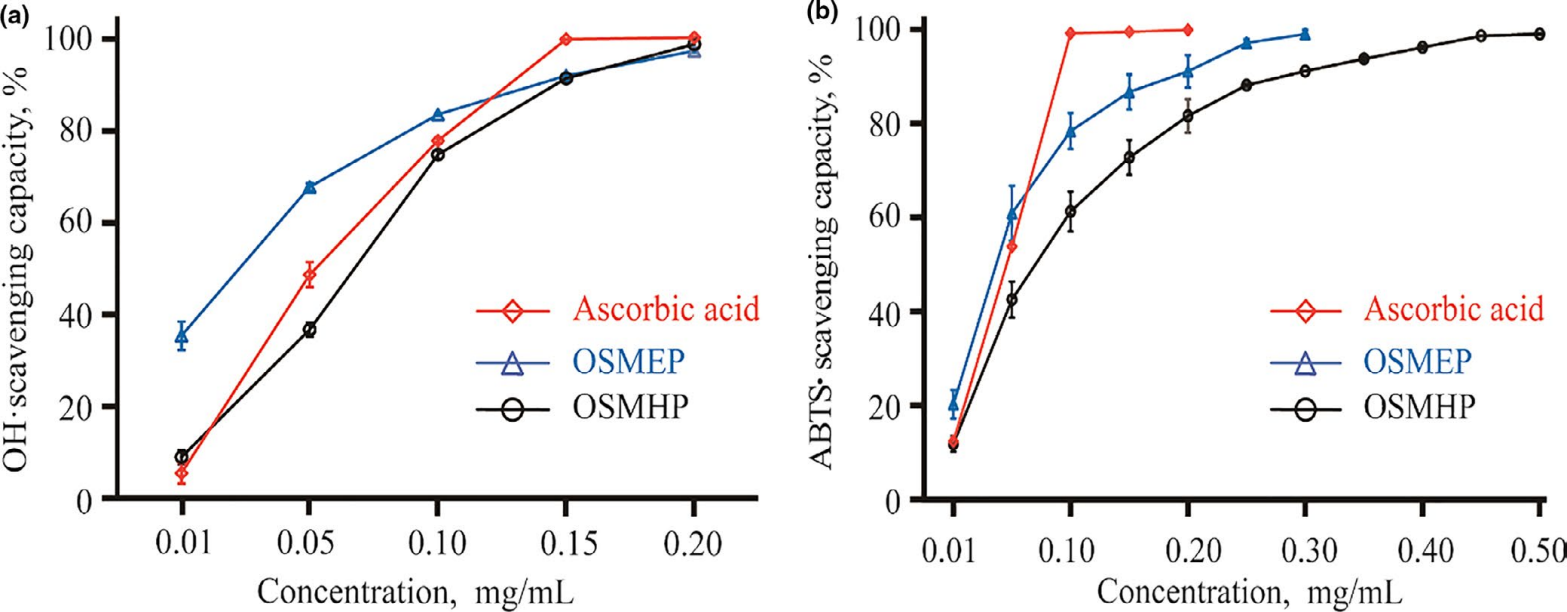
Antioxidant activity analysis in vitro of enzymatically hydrolyzed okra seed meal protein (OSMEP) and hydrolyzed okra seed meal protein (OSMHP). (a) Hydroxyl free radical (OH·) scavenging capacity; (b) ABTS (2, 2‐Azino‐bis (3‐ethylbenzthiazoline‐6‐sulfonic acid)) free radical scavenging capacity. The concentration of OSMEP and OSMHP means the free amino acid nitrogen concentration

OSMEP performed better not only on hydroxyl free radical scavenging capacity (Figure 6a) but also on ABTS free radical scavenging capacity (Figure 6b). Besides, the EC_50_ of OSMEP and OSMHP on hydroxyl free radical scavenging and ABTS free radical scavenging were 0.020, 0.032 mg/ml and 0.053, 0.061 mg/ml, respectively, while the EC_50_ of ascorbic acid on hydroxyl free radical scavenging and ABTS free radical scavenging were 0.048 and 0.034 mg/ml, respectively. The EC_50_ of OSMEP was lower most probably due to the different amino acid composition. Sun (Sun et al., 2012) reviewed that the hydrophobic amino acids promote interaction with free radicals by increasing solubility in lipids; in addition, phenylalanine can combine with free radicals as a proton donor then maintains stability via resonance structure. As can be seen from Table 7, the total hydrophobic amino acid percentage composition of OSMEP was accounted to 25.2%, and it was bit higher than OSMHP which was accounted to 20.0%. Moreover, phenylalanine percentage of OSMEP was 3.6% while it was absent in OSMHP. Furthermore, arginine percentage of OSMEP was 10.9% which was 3.2% higher than that of OSMHP. Studies have confirmed that arginine has strong 2,2‐diphenyl‐1‐picrylhydrazyl (DPPH) free radical and ABTS free radical scavenging ability (Ahmad et al., 2015). The above reasons may lead to OSMEP possesses better antioxidant capacity in vitro than OSMHP.

## 4 CONCLUSION

In this present study, two different hydrolyzing processes were used and optimized, the degree of acidic hydrolysis reached 58.53 ± 1.92%, and the degree of enzymatic hydrolysis was much lower at 16.26 ± 0.56%. Two type of okra seed meal protein hydrolysates were obtained, respectively. The result indicated that okra seed meal can be utilized as a good source for producing protein hydrolysates after lipid extraction. OSMHP can possibly be used for producing HVP which is often applied as a kind of food additive after removal of Chloropropanol. In comparison, with the advantage of better potential antioxidant activity and fuller range amino acid, OSMEP can possibly be used for producing functional food.

During the past decades, plant protein from soybean, wheat, and oil plants was usually used as animal feed and good source of hydrolyzed vegetable protein (HVP). Recently, with the continuous improvement of people's attention to health, interests have emerged in identifying and characterizing bioactive peptides from plant protein since they are rich sources of pharmacological and biological active compounds (Galante et al., 2020). Wang and his colleagues produced bioactive peptides from soybean meal by solid‐state fermentation with lactic acid bacteria and protease (Wang et al., 2014). Peptides prepared from rapeseed meal by solid‐state fermentation were proved to possess antioxidation and memory protection on D‐galactose‐induced memory impairment in aging mice (Ding et al., 2019).

Although there are many researches in the field of utilization of plant protein, there are few researches on acidic and enzymatic hydrolysis of okra seed meal. By this token, the results in this study provide technical support for the expansion and value improvement of okra seed utilization.

## CONFLICT OF INTEREST

The authors declare no conflict of interest.

## ETHICAL APPROVAL

This study does not involve any human or animal testing.
